# Root-to-Shoot Long-Distance Mobile miRNAs Identified from *Nicotiana* Rootstocks

**DOI:** 10.3390/ijms222312821

**Published:** 2021-11-26

**Authors:** Zhuying Deng, Huiyan Wu, Dongyi Li, Luping Li, Zhipeng Wang, Wenya Yuan, Yongzhong Xing, Chengdao Li, Dacheng Liang

**Affiliations:** 1 Hubei Collaborative Innovation Center for Grain Industry, School of Agriculture, Yangtze University, Jingzhou 434023, China; 201572341@yangtzeu.edu.cn (Z.D.); 202071606@yangtzeu.edu.cn (H.W.); 202071603@yangtzeu.edu.cn (D.L.); 201871364@yangtzeu.edu.cn (L.L.); zpwang0813@163.com (Z.W.); 2 Engineering Research Center of Ecology and Agricultural Use of Wetland, Ministry of Education, Yangtze University, Jingzhou 434023, China; 3 Hubei Collaborative Innovation Center for Green Transformation of BioResources, State Key Lab of Biocatalysis and Enzyme Engineering, Hubei Key Laboratory of Industrial Biotechnology, College of Life Sciences, Hubei University, Wuhan 430062, China; wyyuan@hubu.edu.cn; 4 National Center of Plant Gene Research (Wuhan), National Key Laboratory of Crop Genetic Improvement, Huazhong Agricultural University, Wuhan 430070, China; yzxing@mail.hzau.edu.cn; 5 Western Barley Genetics Alliance, Murdoch University, Murdoch, WA 6150, Australia; C.Li@murdoch.edu.au

**Keywords:** long-distance transport, mobile miRNA, root-to-shoot, interfamilial graft

## Abstract

Root-derived mobile signals play critical roles in coordinating a shoot’s response to underground conditions. However, the identification of root-to-shoot long-distance mobile signals has been scant. In this study, we aimed to characterize root-to-shoot endogenous mobile miRNAs by using an *Arabidopsis/Nicotiana* interfamilial heterograft in which these two taxonomically distant species with clear genetic backgrounds had sufficient diversity in differentiating miRNA sources. Small RNA deep sequencing analysis revealed that 82 miRNAs from the *Arabidopsis* scion could travel through the graft union to reach the rootstock, whereas only a very small subset of miRNA (6 miRNAs) preferred the root-to-shoot movement. We demonstrated in an ex vivo RNA imaging experiment that the root-to-shoot mobile *Nb-miR164*, *Nb-miR395* and *Nb-miR397* were targeted to plasmodesmata using the bacteriophage coat protein MS2 system. Furthermore, the *Nb-miR164* was shown to move from the roots to the shoots to induce phenotypic changes when its overexpressing line was used as rootstock, strongly supporting that root-derived *Nb-miR164* was able to modify the scion trait via its long-distance movement.

## 1. Introduction

One of the most fascinating aspects of plant grafting is the movement of macromolecules between a scion and rootstock (e.g., small RNA-mediated long-distance silencing movement [1,2], protein shuttling [3] and genetic material transfer [4,5,6,7,8]). The macromolecule movement may occur locally through cell-to-cell transfer via plasmodesmata (PD) or systematically through vasculature-mediated inter-tissue translocation. There are two specialized vascular tissues, namely the phloem and xylem, that usually serve as the superhighway for the long-distance transport of water, photoassimilates, nutrients, minerals and other signaling molecules. Emerging studies show that macromolecules are also moving through these superhighways. For instance, the florigen signal *FLOWERING LOCUS T (FT)* is generated in leaf tissue but is transmitted to the floral meristem via phloem [9,10,11,12]. This movement is critical for plants to exert their reproductive functions in response to environmental input, which in this case is light. However, new studies have found a certain type of transfer from a scion to rootstock may be physically inconclusive. For example, some proteins that are targeted toward suborganelles are also loaded into phloem and transmitted down to the rootstock, and the chloroplast-localized ferredoxin-NADPH oxidoreductase fused with GFP controlled by the 35S promoter in the scion was found down to the root meristem of a non-transgenic rootstock [13]. Further experiments have found those proteins during their transit to their target suborganelles, rather than via a secreted pathway, can be engulfed into the phloem stream and brought into the rootstock [13], implying that some proteins could be passively transported via phloem during the process of transit.

The passive long-distance transport could also occur to RNA. The majority of the RNAs with abundant expression and longer half-lives were detected as mobile RNAs, and only a small proportion were identified as the low-abundance mobile RNAs, suggesting the passive movement mechanism plays an important role in the intraspecific scion–rootstock exchange [14]. Abundance-driven RNA mobility was also observed in the parasite–host system (e.g., the *Arabidopsis*–*Cuscuta* and tomato–*Cuscuta* interaction) [15]. RNAs in high abundance from either the host or parasite tend to be detected in the tissues from the respective opponent, implying that the movement of highly abundant RNAs might not be selective.

Aside from the abundance model, the structural model provides another mechanism for intercellular RNA movement. A tRNA-like structure is capable of moving between cells, such that the non-mobile *GUS* mRNA can be, when adorned at the 5′ UTR, transmitted to the shoot tip in the grafting assay [16]. These insightful results could spur further investigation on how this particular structure can impart mRNA movement and its underlying mechanisms.

Apparently, the small RNAs, typically 20–24 nt and including small interfering RNA (siRNA) and miRNA [17], do not possess the tRNA-like structure to perform non-autonomous movement. For example, siRNA-mediated post-transcript gene silencing can move from root to shoot or shoot to root without the help of a special RNA structure [1,2,18]. However, the genetic components for small RNA processing, particularly RNA amplification, are required in the recipient tissues (e.g., DICER-like 3 (DCL3) or RNA-dependent RNA polymerase (RDR2)) [1] and also in the sending tissues (e.g., RDR6) [2]. These results further emphasized that some small RNAs could make their move in a quantity-dependent manner.

On the contrary, the endogenous miRNAs are RDR-independent small RNAs (sRNAs) and tend to exert their functions locally [19,20]. However, many studies have shown a good number of miRNAs can perform long-distance movement from a scion to a rootstock [21,22]. We need to point out that so far, the systemically mobile miRNAs have been validated through overexpression of the target miRNA in the scion or examination in a rootstock *hen1* mutant background that is unable to accumulate mature miRNAs. These verifying methods again alluded to the importance of abundance through artificial enrichment of the target miRNAs, thus compromising the biological significance of scion-to-rootstock mobile miRNAs. Questions arise as to whether any miRNA can perform long-distance movement without amplification or phloem streaming or if any miRNA can move from the rootstock to the scion, which is against the phloem stream.

Many studies have used homografts to demonstrate the movement of miRNAs and other macromolecules. However, the conserved sequence in the miRNAs and other RNAs would complicate the distinction of the mobile sequence from the non-mobile. To address the above-mentioned questions and further explore the long-distance macromolecule translocation between the scion and rootstock across the graft union, we used two taxonomically distinct plants, *Arabidopsis thaliana* and *Nicotiana benthamiana*, to construct an interfamilial heterograft [23]. With this system, we demonstrated using small RNA deep sequencing that scion–rootstock communication could be achieved via long-distance mobile miRNAs. Further analysis showed that the mobile miRNAs preferred directional movement and might perform their biological functions in the destination to modify the biological traits.

## 2. Results

### 2.1. Hypocotyl Grafting and Small RNA Deep Sequencing in At Scions and Nb Rootstocks

**Figure 1:**
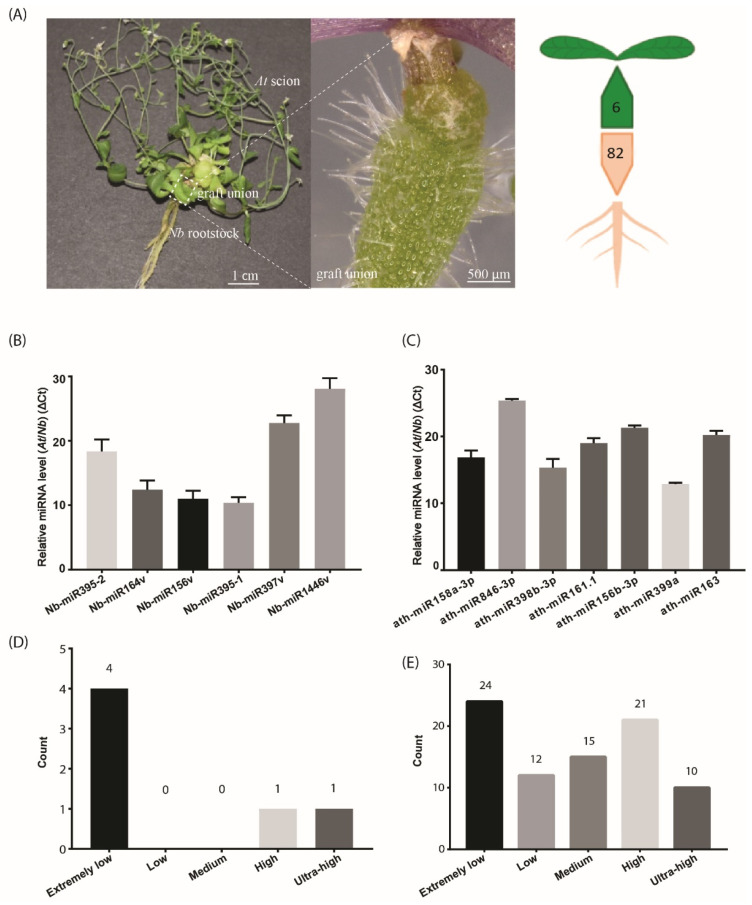
The number of identified mobile miRNAs in the *Arabidopsis/Nicotiana* interfamilial graft. (**A**) A representative *At/Nb* interfamilial graft showing the scion, rootstock and graft union. In total, 6 and 82 miRNAs were identified to move from root to shoot and shoot to root, respectively, in *Nicotiana benthamiana* and *Arabidopsis thaliana*. (**B**) Expression levels of the selected *Nb* miRNAs were determined in the *At* scion by quantitative real-time PCR. (**C**) Expression levels of the selected *At* miRNAs were determined in the *Nb* rootstock by quantitative real-time PCR. (**D**) Classification of expression level for the identified mobile miRNA in the scion. (**E**) Classification of expression level for the identified mobile miRNA in the rootstock. Data in (**B**,**C**) (mean ± standard deviation) were generated from three biological replicates and two technical replicates for each. U6 RNA was used as the internal reference. ΔCt values were the difference between the Ct values of the selected miRNA and U6 and thus were inversely proportional to the amount of the target miRNA in the samples.

To stringently characterize the small RNAs that move from root to shoot, we adopted hypocotyl grafting, in which the graft union occurred at the hypocotyl region of *At* and *Nb* (Figure 1A). This type of graft, unlike those exploring the source-to-sink movement, separated the shoot and root spatially, thus avoiding the leaf source-derived signals being included. We found that the life span was extensively extended in the *At/Nb* grafts [23], and we hypothesized that any small RNAs from the rootstock could partially represent contributing factors to the observed phenotypic changes. With this premise, we collected the samples at the mature stage of the *At/Nb* graft or 90 days after grafting (DAG) for small RNA library construction. Because sample collection in a similar timescale is not applicable for *At/At* or *Nb/Nb* self-grafts, we chose the similarly developmental stage of self-grafts (e.g., 40–45 DAG) for a negative control.

Four groups of materials that were labeled as AGS (*Arabidopsis* grafting scion in *At/Nb* heterograft), ACS (*Arabidopsis* control scion in *At/At* homograft), NGR (*Nicotiana* grafting rootstock in *At/Nb* heterograft) and NCR (*Nicotiana* control rootstock in *Nb/Nb* homograft) were harvested for RNA deep sequencing. More than 13.85 million clean reads in the size of 18–30 nt for each sample were obtained by removing the low-quality reads (e.g., reads of a length <18 nt or >30 nt, reads with more than three unknown bases or reads without adaptors) (Table S1). Bowtie analysis was performed to determine the read distribution in the clean reads. The results showed that the unannotated reads accounted for more than 50% of all reads in all samples, and the miRNA reads accounted for about 6.3%, 4.4%, 1.9% and 2% in the AGS, ACS, NGR and NCR, respectively (Figure S1). More than 60% of the total sRNA reads including miRNA and unannotated RNAs in the AGS and ACS were further mapped to the *Arabidopsis* genome, and less than 1% of the remaining unmapped reads were re-mapped onto the *Nb* genome (Table 1). Similarly, in the *Nb* rootstock, about 0.29% and 0.05% of the total sRNA reads from NGR and NCR, respectively, were mapped to the *Arabidopsis* genome (Table 2). These results suggested that a small fraction of the total small RNAs could potentially move across the graft union.

### 2.2. Small RNA Movement across the Graft Union

The overall identified potential mobile miRNAs are listed in Tables S2 and S3. A very simple method to recover the high-confidence mobile sRNAs dictates that RNA reads from either the scion or rootstock that are mapped to the opposite part of the grafts should not appear in the corresponding part of self-grafts. Under this stringent purview, we recovered six candidates from *Nb* in the *At* scions (Table 3). These miRNAs were highly conserved, belonging to five miRNA families, including *miR156*, *miR164*, *miR395*, *miR397* and *miR1446*. Since these miRNAs showed sequence variation to the existing miRNA members, they were referred to as *Nb-miR156v*, *Nb-miR164v*, *Nb-miR395-1*, *Nb-miR395-2*, *Nb-miR397v* and *Nb-miR1446v*, respectively, in this study (Table 3). The precursor sequences were also identified from the *Nb* genome (Table S4). They may be ascribed as high-confidence root-to-shoot mobile candidates, as they repeatedly appeared in all heterografting samples.

Likewise, the same selection criteria applied to the *Nb* rootstock led to the identification of 82 *At* miRNAs (Table S3), a much higher number than those in the rootstock-to-scion migrating direction. This in turn agreed with the bulk flow in the source-to-sink direction. These potentially mobile miRNAs accounted for nearly one quarter of the total *Arabidopsis* miRNAs (miRbase), suggesting the movement of substantial miRNAs via the phloem. We further compared the miRNAs in the AGS samples with those in the *Nb* samples and found 31 *At* miRNAs that shared the exact sequence with those in the *Nb* (Table S5). Thus, we could not determine their mobility in this study. This might further imply that the number of mobile miRNAs through phloem could potentially increase with an improved technique.

We also noticed that the two subsets of mobile miRNAs shared no essential overlaps. Although the *miR156* family member appeared in both subsets (Table 3 and Table S3), the *Nb miR156* sequence corresponded to the sense strand, while the *At miR156* sequence recovered in the rootstock was located in the antisense strand, agreeing with previous studies showing that the miR156* strand could be detected in the phloem [24]. Taken together, the non-overlaps between the two mobile miRNA subsets implied the divergence of the regulatory mechanisms of the top-down and down-top miRNA movement.

### 2.3. Mobile miRNA Detection

To detect the mobile miRNAs, we adopted the stem-loop RT-qPCR procedure which proved to be specific and sensitive to miRNA detection [25]. In the scion tissues, five out of the six selected miRNAs were detected (Figure 1B), including *Nb-miR395-1*, -2, *Nb-miR164v*, *Nb-miR156v, Nb-miR397v* and *Nb-miR1446v* (primers in Table S6). In the rootstock, the seven selected miRNAs from *At* were detected (Figure 1C). All these detected miRNAs showed a very low accumulation relative to the U6 reference. We further checked the expression of these mobile miRNAs in the non-grafting tissues and found nearly one third of the root-to-shoot miRNAs (29%) belonged to the extremely low expression group (Figure 1D). In the shoot-to-root miRNA subset, nearly one third of them belonged to the extremely low expression group, and around 38% of the miRNAs were from the high or ultra-high expression group (Figure 1E), suggesting the abundance model [14] could partially explain the disparity in the miRNA numbers between the two subsets, given that the high expression miRNAs could easily be brought down to the rootstock through the phloem bulk flow.

### 2.4. Pre- and Mature miRNA, but Not the Pri-miRNA, Can Be Detected in the Scion or Rootstock

**Figure 2:**
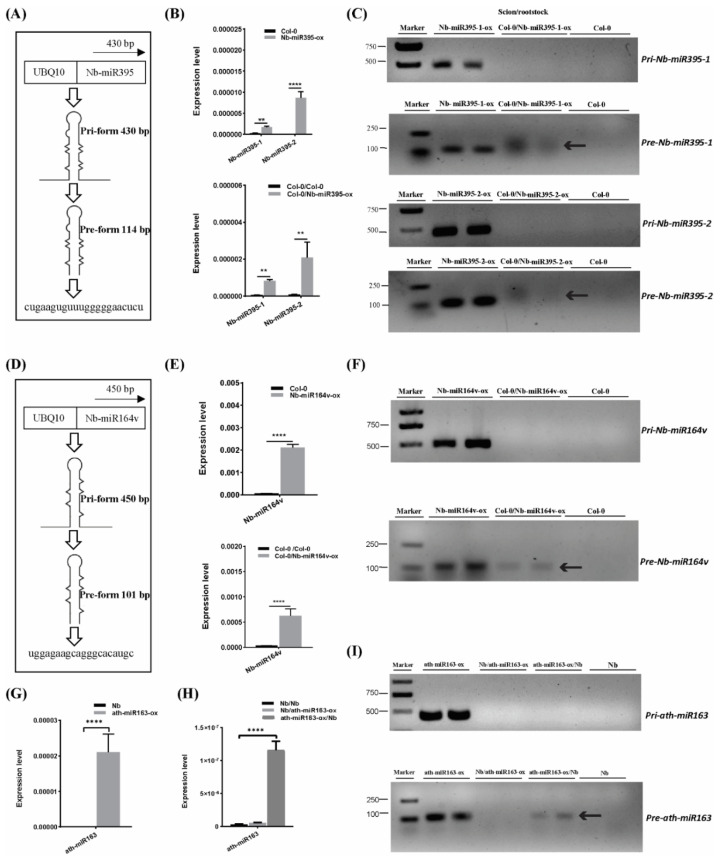
Pre- and mature miRNA detection in the scion. (**A**) Pri and pre forms of *Nb-miR395-1*. (**B**) *Nb-miR395-1, -2* detection in the *Arabidopsis* overexpressing line (top) and in the WT Col-0 scion (bottom). (**C**) RT-PCR assays on pri and pre forms of *Nb-miR395-1, -2*. (**D**) Pri and pre forms of *Nb-miR164v*. (**E**) *Nb-miR164v* detection in the *Arabidopsis* overexpressing line (top) and in the WT Col-0 scion (bottom). (**F**) RT-PCR assays on pri and pre forms of *Nb-miR164v*. (**G**) QPCR quantification of *ath-miR163* in the *Nb* overexpressing line. (**H**) QPCR quantification of *ath-miR163* in the WT *Nb* scion and rootstock. (**I**) RT-PCR assays on pri and pre forms of *ath-miR163* in the WT *Nb* scion and rootstock. The arrow indicates the precursor transcript in the WT scion or rootstock. The bars represent the means and standard deviations of six replicates (three biological replicates, each with two technical replicates). The two asterisks indicate *p* < 0.01, and four asterisks indicate *p* < 0.0001 (*t*-test).

To further confirm the mobility of these miRNAs, we overexpressed the pri-miRNA coding sequence of *Nb-miR395-1, Nb-miR395-2* and *Nb-miR164v* in *Arabidopsis* to check whether they sustained their mobility in *Arabidopsis* (Figure 2A,D). The stem-loop QPCR results revealed that the mature miRNAs were expressed in *Arabidopsis* and could be detected in the scion tissues in which the miRNA-overexpressing *Arabidopsis* was used as the rootstock (Figure 2B,E), suggesting the mature *Nb-miR395-1, Nb-miR395-2* and *Nb-miR164v* could move to the scion as shown in the heterografts (Figure 1B). We found that the transcribed pri-miRNAs in all these selected miRNA loci (*Nb-miR395-1, Nb-miR395-2* and *Nb-miR164v*) were beyond detection in the scions (Figure 2C,F), but the pre-miRNAs could be detected in the *Arabidopsis* scions (two independent materials), suggesting the pre-miRNAs together with the mature miRNAs could be transferred from the roots to the shoots. To further evaluate the specificity of the miRNA movement, we overexpressed an *Arabidopsis*-specific miRNA gene, *miR163*, which showed a potential for rootward mobility (Table S3) in *N. benthamiana* and used this transgenic *Nb* line (*ath-miR163-ox*) as the scion or rootstock (Figure 2G–I). QPCR showed that *miR163* could not be detected in the wild-type (WT) *Nb* background but was highly expressed in the *Nb* transgenic line (Figure 2G). The grafting experiment revealed that *miR163* from the *Nb* overexpressing line could be detected in the WT *Nb* rootstock rather than the *Nb* scion (Figure 2H), suggesting the specificity of its directional movement. Likewise, the pre-miRNA (but not the pri-miRNA) of *miR163* could be detected in the WT *Nb* rootstock (Figure 2I), supporting the mobility of the *miR163* precursor.

### 2.5. Validation of miRNA Movement Using the MS2 System

**Figure 3:**
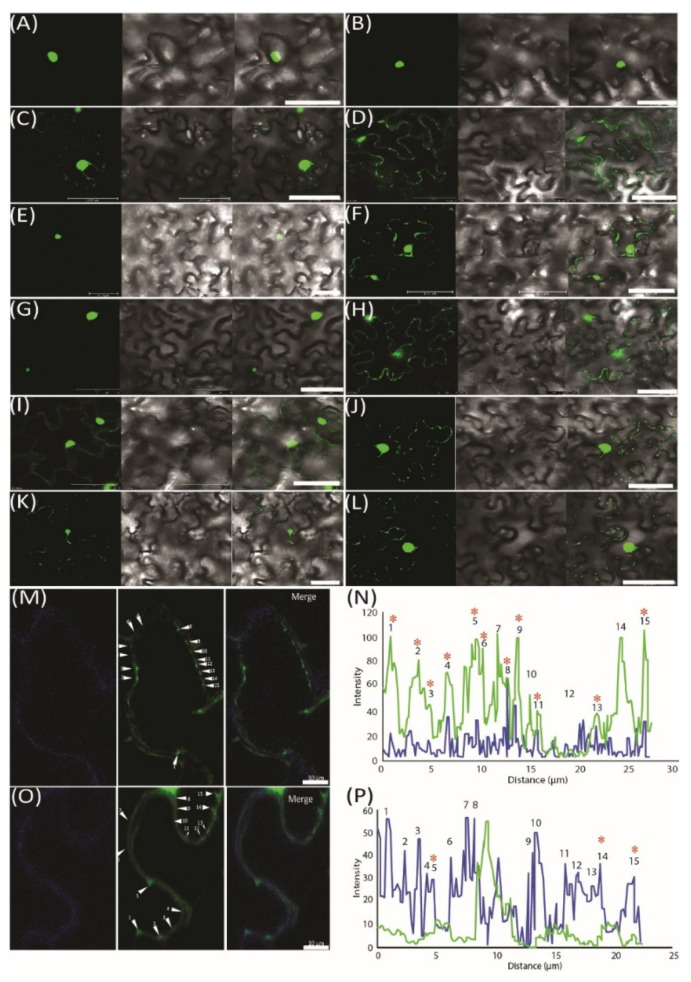
Pre form (rather than pri form) of root-to-shoot mobile miRNAs that were targeted toward plasmodesmata (PD). (**A**) MS2_FD_-GFP localization in the nucleus of *Nb* epidermal cells 2 days after *Agrobacterium* infiltration. (**B**) Co-infiltration of MS2_FD_-GFP and an SL_24_ empty vector. (**C**) Co-infiltration of MS2_FD_-GFP and an *Actin2-SL_24_* negative control. **(D**) Co-infiltration of MS2_FD_-GFP and SL_24_-FT (*FLOWERING LOCUS T*) used as a positive control. (**E**) *Pri-Nb-miR395-1* co-expressed with MS2_FD_-GFP. (**F**) *Pre-Nb-miR395-1* co-expressed with MS2_FD_-GFP. (**G**) *Pri-Nb-miR397v* co-expressed with MS2_FD_-GFP. (**H**) *Pre-Nb-miR397v* co-expressed with MS2_FD_-GFP. (**I**) Pri-*Nb-miR164v* co-expressed with MS2_FD_-GFP. (**J**) Pre-*Nb-miR164v* co-expressed with MS2_FD_-GFP. (**K**) Mature *Nb-miR164v* fused with SL_24_ and its co-expression with MS2_FD_-GFP. (**L**) Three tandem repeats of *Nb-miR164v* (3x*Nb-miR164v*-SL_24_) fused with SL_24_ and its co-expression with MS2_FD_-GFP. (**M**) Co-expression of *3xNb-miR164v-SL_24_* and MS2_FD_-GFP in aniline blue-stained *N. benthamiana* leaves. Left: image taken in the aniline blue channel. Middle: image taken in the GFP channel. Right: merged image from left and middle image. (**N**) Co-localization analysis of the GFP foci in *3xNb-miR164v-SL_24_* transiently expressing leaves. (**O**) Co-expression of non-mobile *Actin2-SL_24_* RNA and MS2_FD_-GFP in aniline blue-stained *N. benthamiana* leaves. Left: image taken in the aniline blue channel. Middle: image taken in the GFP channel. Right: merged image from left and middle image. (**P**) Co-localization analysis of the GFP foci in *Actin2-SL_24_* transiently expressing leaves. The numbers in (**M**–**P**) represent individual GFP spots. Co-localization of the GFP signal with aniline blue is indicated by a star sign. Scale bar in (**A**–**L**) represents 50 µm and scale bar in (**M**,**O**) represents 10 µm.

To further demonstrate the movement of miRNAs, we used an MS2 RNA visualizing system, which was adopted to visualize the RNA movement in the plants [26]. MS2_FD_-GFP was only detected in the nucleus when transiently co-expressed with the nonmobile RNA *Actin2-SL_24_* [27] or SL_24_ blank vector (Figure 3A–C). To test how the root-to-shoot miRNAs could be mobile between cells, we fused two different forms of miRNAs (i.e., the pri and pre forms) with SL_24_. As seen in Figure 3E–G, the full-length pri forms of *Nb-miR395-1* and *Nb-miR397v* were mainly accumulated in the nucleus, giving out a similar pattern to that of MS2_FD_-GFP or that of nonmobile RNAs (Figure 3A–C,E,G). However, the pre forms of *Nb-miR395-1* and *Nb-miR397v* were localized to the punctate foci around the cell periphery (Figure 3F,H) in a pattern similar to that of *FLOWERING LOCUS T (FT)* mRNA *FT-SL_24_* (Figure 3D). In pri-miR164-SL_24_, the GFP signal was simply uniformly detected at the cell periphery (Figure 3I). However, in pre-*Nb-miR164v*-SL_24_, the strong signal of the punctate green fluorescent foci appeared around the cell periphery (Figure 3J). Similarly, the mature *Nb-miR164v* and three tandem repeats of *Nb-miR164v* (3x*Nb-miR164v*-SL_24_) could also be located in the punctate foci (Figure 3K,L), agreeing with the miRNA detection results (Figure 2E,F). To further confirm the punctate foci were overlapping with PD, we examined the localization of 3x*Nb-miR164V*-SL_24_ GFP fluorescence and the signal of aniline blue that was used to indicate callose deposition at the PD neck, and we found the two signals were co-localized in the majority of the examined foci (Figure 3M,N). In contrast, the *Actin2-SL_24_* GFP signal, shown in Figure 3O,P, was very scarce and only partly overlapping with the aniline blue signal (3 of 15 stains). These findings suggest that the mature and pre forms of the selected miRNAs were mobile and most probably targeted to PD to be transferred to the adjacent cell.

### 2.6. Phenotypic Modification in the Scion by Root-to-Shoot Mobile Nb-miR164v

**Figure 4:**
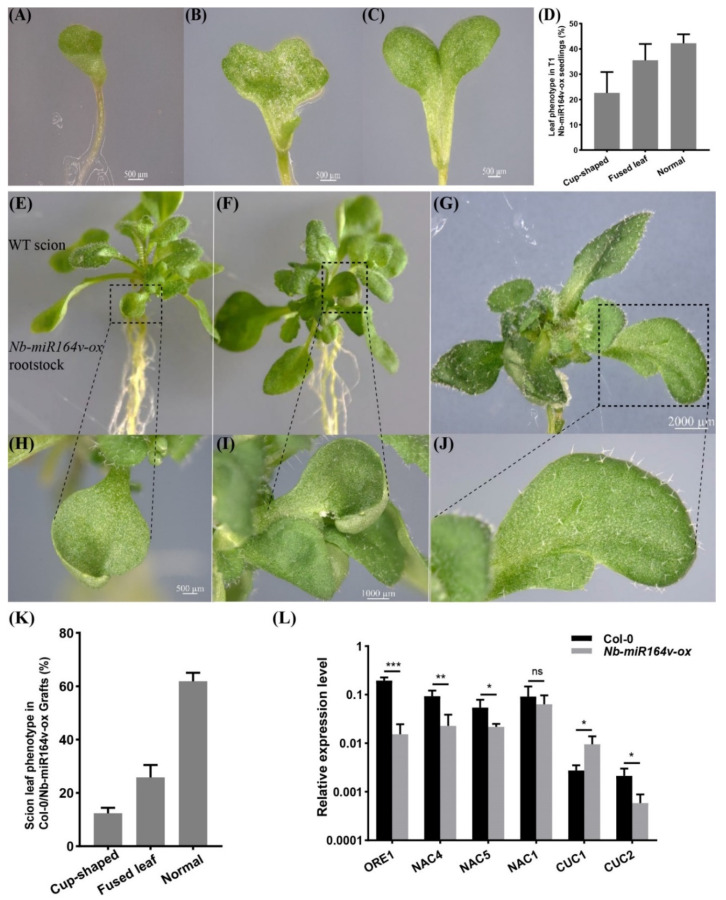
Phenotypic changes in a scion by root-to-shoot transmissible *Nb-miR164v*. (A–D) Overexpression of *Nb-miR164v* driven by a ubiquitin10 promoter in *Arabidopsis*, resulting in leaf defects such as the cup-shaped cotyledon (**A**), fully fused cotyledon (**B**) and partially fused cotyledon (**C**). (**D**) The percentage of *Nb*-*miR-164v-ox* seedlings showing the leaf phenotype as in (**A**–**C**). The bars represent the means and standard deviations of the two experiments (*N* = 50 in each experiment). (**E**–**G**) The WT *Arabidopsis* was used as a scion and grafted to the above plants with leaf defects. (**H**–**J**) Cup-shaped and partially fused leaves from the scion. Note that not all the leaves from the scion showed defects. (**K**) The percentage of grafts showing the altered leaf phenotype. The bars represent the means and standard deviations of three experiments (*N* = 15 in each experiment). (**L**) Expression of *Nb-miR164v* targets in the scion leaves. The bars represent the means and standard deviations of six replicates (three biological replicates, each with two technical replicates). *, **, and *** indicate *p* < 0.05, <0.001, and <0.0001, respectively.

We next asked whether these root-to-shoot mobile miRNAs could cause any biological consequences on the scion morphology after moving into the scion. For this, we overexpressed *Nb-miR395-1, Nb-miR395-2, Nb-miR397v, Nb-miR1446v* and *Nb-miR164v* in *Arabidopsis* to check if they could cause any phenotypic changes. In the T2 generation of the *Nb-miR395-1, Nb-miR395-2, Nb-miR397v* and *Nb-miR1446v* overexpressing lines, no visual phenotype or developmental defects were observed (Figure S2). The grafting experiment involving these lines as rootstocks did not show any observable phenotypic difference with the Col-0 self-grafts (Figure S2). As a representative example, *Nb-miR164v* was chosen for further investigation, since the overexpression of *Nb-miR164v* in *Arabidopsis* gave rise to copious morphological defects, such as the fused and misshapen leaves (Figure 4A–D). These phenotypes were exactly similar to the *Arabidopsis*‘s own *miR164* overexpressing lines [28,29,30], suggesting the similar role of *Nb-miR164v* in leaf development.

We then grafted the *Nb-miR164v* overexpressing line as a rootstock with the WT Col-0 scion. The first emerging leaves in these grafts showed the typical defects (Figure 4E–J), and the fusion of two different leaves could also occur (Figure 4G,J). In total, around 38% of the grafts showed the typical leaf phenotypes (Figure 4K). Since these defects are characteristic for plants with reduced CUC1 or CUC2 activity [28,29,30], we quantified the levels of the *miR164* targets in the WT and the grafts. The results showed that the majority of the *miR164* targets were significantly downregulated in the WT scion (Figure 4L), which was consistent with the expectations. These results strongly indicated that the *Nb-miR164v* in the rootstock moved into the scion to modify the leaf shape.

## 3. Discussion

### 3.1. Phloem-Mediated Bulk Flow of Mobile RNAs

Macromolecules are able to carry out their function in a non-autonomous way. This phenomenon has become increasingly appreciated for their roles in intercellular and inter-tissue communication by using a grafting technique [31,32,33]. By such means, Notaguchi et al. [34] identified 138 mobile mRNAs by constructing a graft between *At* and *Nb* at the bolting stage. A comparative number of mobile transcripts (183 *Nb* transcripts) was identified to move into a tomato rootstock using an *Nb*/tomato heterograft system [35]. Recently, with the long-read RNA-seq technology, Li et al. [36] were able to detect a very small subset of mRNAs moving between the heterografting partners involving soybean and common bean (163 mRNAs from soybean and 129 mRNAs from common bean), but with only around 56% of each subset being full-length coding sequences (91 from soybean and 77 from common bean). Aside from that, this study identified 100 miRNAs that were predominantly produced in shoots and transported to the roots. Our results also showed a comparable number of miRNAs (82 miRNAs) moving down to the rootstock in the *At/Nb* heterograft at 90 DAG, implying the common regulatory mechanisms for miRNA movement. In addition, many miRNAs moving from scion to rootstock have been reported before. For example, the well-documented shoot-to-root mobile sRNA *miR399* [37,38,39,40] was recovered in the samples. Meanwhile, several miRNAs, such as *miR156* [41,42,43], *miR167* [41,42,44,45], *miR168* [39], *miR169* [39], *miR172* [42,45], *miR829* [39], *miR2111* [40,43,46] and *miR403* [39,44], that were phloem-mobile sRNAs were also found in the *Nb* rootstock, particularly *miR2111*, which was recently confirmed to move from shoot to root to regulate nodulation in soybean [46].

Interestingly, the number of mobile mRNA transcripts seemed to be vastly increased in the homograft or closely related species. For example, there were about 2006 RNA transcripts transported between 2 *At* genotypes [27], more than 3000 mobile mRNAs across the graft union in 2 grapevine species [47] and more than 3500 mRNAs exchanged between cucumber and watermelon [48]. A discrepancy of this magnitude could arise from the process of vascular connection between the scion and rootstock; that is, the phloem connection in the grafts involving closely related species performed much better than in those distantly related species, thus allowing more RNA transcripts to move into the sink tissues through the phloem. For example, phloem translocation of [^14^C]-sucrose could occur 5–10 days after grafting in autografts and in closely related grafting partners involving *Lycopersicon* and *Solanum* (compatible combination) but not in the *Vicia/Helianthus* heterograft (less compatible combination), and a strong correlation between phloem connection and assimilate transport across the graft interface could be found [49]. Similarly, [^14^C]-sorbitol translocation in the incompatible pear/quince heterograft was greatly reduced compared with the compatible graft or the autograft [50]. Given that the majority of mobile RNAs move through the phloem by bulk flow, the number of identified mobile RNAs could strongly rely on the phloem connection between the scion and rootstock.

Compared with the shoot-to-root mobile RNAs, the number of root-to-shoot mobile RNAs was extremely low. In our case, there were only six miRNAs being detected (Figure 1A). The immediate question that arises is how these miRNAs can be transmitted to the shoot, given that they are moving against phloem streaming. One possible route is the cell-to-cell transmission from the bottom, as shown in long-distance mobile silencing [2]. We indeed showed that the *NbmiR395, 397* and *164* could be transported through PD (Figure 3), which may constitute the first step of shootward non-cell-autonomy. Because the cell-to-cell transmission of miRNA usually operates locally to form a gradient for its function [51], the graft-transmissible long-distance movement of miRNA could hardly be achieved simply through local transmission. That aside, miRNA movement is an amplification-independent process, and thus root-derived miRNA movement into shoots warrants further explanation.

Another seemingly remote possibility could be attributed to xylem transport. Given the root-to-shoot transport against phloem streaming, the possibility for bottom-to-top transport via xylem might not be irrefutably excluded, as several studies have observed before. For instance, the *StBEL5* RNA can be detected in the micro-dissected xylem in the potato [52], and RNA profiling in xylem tissue also identifies many non-coding RNAs and mRNAs [53,54,55]. Furthermore, small peptides can signal their physiological status to the shoots by traveling through xylem [56]. Nevertheless, Buhtz et al. [42] found no RNAs in the xylem sap of *Brassica*, apparently contradicting the results mentioned above. This contradiction could come from a technological difference and could be resolved through technique improvement, particularly the improvement of xylem isolation while maintaining RNA integrity.

### 3.2. Robustness of Heterografts in the Identification of Root-to-Shoot Long-Distance Mobile miRNA

Bioinformatic analysis of the mobile RNA dataset indicated that smaller transcripts tend to be more mobile [14]. Thus, it is reasonable to infer that small RNA (including miRNAs) should have a high tendency to move. Indeed, more and more studies have shown that many small RNAs are mobile [36,57,58]. However, the identification of bottom-to-top mobile miRNAs has been lacking. In this study, we have identified six candidates that can move from rootstock to shoot (Table 3). Among them, *miR395* and *miR397* are very likely possessing the property of root-derived signals: highly or exclusively expressed or inducibly expressed in the root but partially accumulated in the shoot. Taking the *Arabidopsis* data as an example, the promoter activity of *miR395a* and *miR395b*, *miR395c* and *miR395d* indicates that they are highly induced in the *Arabidopsis* roots [59]. Since *miR395* regulates sulfate accumulation and allocation in *Arabidopsis* [60], the expression pattern of the *miR395* family implied that root-to-shoot movement might represent a physiologically relevant event in which the sulfate availability sensed by the roots would coordinate the nutritional redistribution in the shoot by emanating root-derived functional macromolecules (e.g., *miR395* in this case) to regulate the sulfate-associated metabolic networks.

Similarly, *miR397* also showed the above property. The expression of *miR397* was strongly induced in the Cd-treated roots but only partially elevated in the shoots [61], a very promising sign for the root-derived signal for regulating shoot response. *miR397* is also induced by other stress stimuli, such as H_2_O_2_ [62] and heat stress [63,64]. Since our previous study also showed that H_2_O_2_ plays critical roles in regulating root-to-shoot mobile silencing [65], the correlation of *miR395* expression with cellular H_2_O_2_ or other stressor-induced H_2_O_2_ fluctuations could reflect its root-to-shoot mobility.

### 3.3. Mobile Small RNA and Trait Modification in Grafts

Grafting-induced phenotypic changes have been widely documented [66,67,68]. Various traits, such as the flowering time, plant architecture, fruit flavor and quality, as well as the response to biotic and abiotic stress, can be modified by grafting. The mechanisms of underlying scion or rootstock phenotypic changes could be very diverse and have been extensively explored, with the focus on what signals communicate between the scion and rootstock to have their impact on phenotypes [67,69]. Grafting experiments combined with next-generation sequencing technology and genetic studies have shown that many sRNAs, particularly the 23–24-nt siRNAs associated with epigenetic regulation in the scion, moved into the rootstock to induce DNA methylation in the target loci [20,70,71]. In terms of scion trait modification, one recent study showed that an enhanced growth vigor conferred by the *msh1* rootstock in both *Arabidopsis* and tomato graft progenies was due to RdDM-dependent epigenetic changes in 1380 differentially methylated genes, including many auxin-related gene pathways [72].

The data from this study showed that several miRNAs could move from rootstock to scion, and we further found that *miR164* could deliver a phenotypic impact on scion leaf development (Figure 4). Actually, *miR164* is mainly expressed in the roots (and almost absent in the leaves of *Arabidopsis*) to regulate the root morphology through the *miR164*-*NAC1* module [73], suggesting the property of root-derived signals for *miR164*. Interestingly, further study shows that it also regulates the leaf senescence through the *miR164*-*ORE1* module in the leaf [74]. Given the mobility of *miR164* from rootstock to scion identified in this study, it is very tempting to speculate that root development could be coordinated with leaf development via the long-distance movement of *miR164*. Further experiments need to be designed to reveal this potential relationship.

## 4. Materials and Methods

### 4.1. Plant Material and Growth Conditions

Seeds of the wild-type (WT) *Arabidopsis thaliana* (Col-0) and the WT *Nicotiana benthamiana*, as well as transgenic lines including *Nb-miR395-1, -2, Nb-miR397v* and *Nb-miR164v* overexpressing *Arabidopsis* lines and ath-miR163 overexpressing *Nb* lines, were surface-sterilized in chlorine gas for 1 h and then plated on sterile Murashige and Skoog (MS) medium supplemented with 3% (*w*/*v*) sucrose. The plants were grown vertically under long day conditions (16 h light, 8 h dark, 22–23 °C).

### 4.2. Grafting

The micro-grafting procedure was essentially described in detail by Andersen et al. [75]. Briefly, the *Nb* young seedlings that were no more than 8 mm long were used as rootstocks, and the *At* seedlings that were 2 cm long were used as scions. The cut was made halfway from the base of each hypocotyl, and the cut surfaces were pushed against each other with a certain tension. The grafts were grown on moisturized Whatman paper for 2 days, and then the grafts were gently lifted with forceps and placed vertically on the MS medium with 1% agar and 3% sucrose (*w*/*v*) in the growth room (16 h light, 8 h dark) at 22–23 °C.

### 4.3. Tissue Collection and RNA Extraction

Ninety DAG grafts were used for each tissue collection. Four different types of tissues were collected from an *At/Nb* heterograft and a self-graft control (*At/At* and *Nb/Nb*), which were the scion samples of AGS (*At* grafting shoots) and ACS (*At* control shoots) and rootstock samples of NGR (*Nb* grafting roots) and NCR (*Nb* control roots). For the aerial parts, the old and yellowing leaves, including rosette leaves, were removed. The stems, cauline leaves and flowers were harvested. For the root tissue, we collected the whole *Nb* roots, excluding the hypocotyl part of the graft union. Each sample was approximately 200 mg and was immediately frozen in liquid nitrogen and stored at −80 °C in a freezer. Three independent biological samples for each type were harvested. The total RNA was obtained using the TRIzol reagent according to Invitrogen’s instructions. The RNA concentration was measured using NanoDrop 2000 (ThermoFisher Scientific, Waltham, MA, USA).

### 4.4. Small RNA Sequencing and Analysis

The RNA quantity and integrity were measured with Qubit 2.0 (Life Technologies, ThermoFisher Scientific, Waltham, MA, USA) and a 2100 Bioanalyzer (Agilent Technologies, Santa Clara, CA, USA), respectively. A small RNA library was prepared using a Next Ultra small RNA Sample Library Prep Kit for Illumina (New England Biolabs, Ipswich, MA, USA). The total RNA was ligated with a 5′ and 3′ adapter sequentially and then reversely transcribed into cDNA. After cDNA PCR amplification and gel purification, the insert size and quantity of the constructed library were assessed with a 2100 Bioanalyzer (Agilent Technologies, Santa Clara, CA, USA) and Qubit 2.0 (Life Technologies, ThermoFisher Scientific, Waltham, MA, USA), respectively. The sequencing procedure was performed with an Illumina HiSeq Xten system at BioMarker Technologies in Beijing, China. The total sRNA reads, including miRNAs and unannotated RNAs, were then obtained by removing sequences from other non-coding RNAs, such as rRNA, tRNA, snRNA and snoRNA in a repetitive sequence using the bowtie tool to align against the Silva, GtRNAdb, Rfam and Repbase databases.

### 4.5. miRNA Detection

Stem-loop RT-qPCR was used to quantify the mobile miRNAs. The stem-loop RT primers and forward qPCR primers were designed using miRNA Design V1.01 software (Vazyme Biotech, Nanjing, China). The first cDNA was synthesized from 1 µg of the total RNA using an miRNA 1st Strand cDNA Synthesis Kit (Vazyme, MR101, Nanjing, China) following the manufacturer’s instruction. The target miRNA was detected by QPCR using the kit’s miRNA Universal SYBR qPCR Master Mix (2×) containing mQ primer R (Vazyme, MQ101, Nanjing, China). Each QPCR reaction was performed in a volume of 20 µL containing 1 µL cDNA, 0.4 µL of each forward qPCR primer (10 µM), 0.4 µL of mQ primer R (10 µM) and 10 µL of 2× miRNA Universal SYBR qPCR Master Mix (Vazyme, MQ101, Nanjing, China). The nuclear small RNA U6 was used as the internal reference gene [76]. QPCR was performed on a Bio-Rad CFX cycler, and the cycling conditions were as follows: 5 min at 95 °C, followed by 40 cycles at 95 °C for 10 s and 60 °C for 30 s. All reactions were run in biological triplicate. A list of the stem-loop and qPCR primers used in this study is given in Supplementary Table S6.

### 4.6. RT-PCR on Grafted Arabidopsis and Nicotiana Transgenic Lines

Three biological replicates of the grafting samples were used to test the mobile transcripts. The total RNA was extracted from the aerial parts (excluding rosette leaves) at 30 DAG using TRIzol reagent. The RNA was quantified with a NanoDrop 2000 spectrophotometer and then pretreated with gDNase to remove any DNA. The 1st strand cDNA from 1 µg RNA was synthesized in a 20-µL volume using a FastQuant RT Kit (TIANGEN, Beijing, China). Then, 1 µL cDNA was used for PCR with 40 cycles. The primers for Figure 2C,F,I are provided in Supplementary Table S6.

### 4.7. Plasmid Construction

The pri- and pre-sequence of *Nb-miR395-1, -2, Nb-miR397v* and *Nb-miR164v* were amplified from *Nb* genomic DNA using the corresponding primers shown in Supplementary Table S6. These amplified genomic DNA fragments were then purified and cloned downstream of ubiquitin 10 promoter in a binary vector p2S3CherryUniH and then confirmed by Sanger sequencing. All plasmids were then transformed into *Agrobacterium tumefaciens* strain GV3101. The transgenic plants were selected using 50 mg/L hygromycin.

The precursor and primary sequences of miRNAs *Nb-miR395-1, Nb-miR397v* and *Nb-miR164v* were amplified from *Nb* genomic DNA using primers Nb-miR395-1-SL_24_-F1, Nb-miR395-1-SL_24_-R1, Nb-miR395-1-SL_24_-F2, Nb-miR395-1-SL_24_-R2, Nb-miR397-SL_24_-F1, Nb-miR397-SL_24_-R1, Nb-miR397-SL_24_-F2, Nb-miR397-SL_24_-R2, Nb-miR164-SL_24_-F1,Nb-miR164-SL_24_-R1,Nb-miR164-SL_24_-F2 and Nb-miR164-SL_24_-R2 (Table S6). A 332-bp fragment of an *Actin2* gene was amplified using primer AT-Actin2-SL_24_-F and AT-Actin2-SL_24_-R. A partial FT cDNA fragment corresponding to the mobile part [77] was amplified from *Arabidopsis* cDNA using primer FT-SL_24_-F and FT-SL_24_-R (Table S6). The amplified sequences were purified and cloned into the MfeI-linearized pUbik-SL_24_ vector individually and confirmed by sequencing. All constructs were transformed into *Agrobacterium tumefaciens* strain GV3101.

For miR164 tandem repeat construction, 100 bp of a partial GUS fragment fused with an *Nb-miR164v* mature sequence was amplified using primers Nb-miR164 P1 and Nb-miR164 P8 (1 repeat). The other fragments were amplified with the following primers: Nb-miR164 P1, Nb-miR164 P2, Nb-miR164 P3, Nb-miR164 P4, Nb-miR164 P5 and Nb-miR164 P8 (3 repeats), as well as Nb-miR164 P1, Nb-miR164 P2, Nb-miR164 P3, Nb-miR164 P4, Nb-miR164 P5, Nb-miR164 P6, Nb-miR164 P7 and Nb-miR164 P8 (4 repeats) (Table S6). After BsaI digestion, these fragments were ligated by T4 ligase (NEB). We then obtained the Nb-miR164 repeat fragment by PCR amplification with primers Nb-miR164 P1 and Nb-miR164 P8. The amplified fragment was purified and cloned into the MfeI-linearized pUbik-SL_24_ vector and then confirmed by Sanger sequencing. All plasmids were then transformed into *Agrobacterium tumefaciens* strain GV3101.

### 4.8. Infiltration and Confocal Microscopy

The above constructs were individually transformed into *Agrobacterium tumefaciens* strain GV3101. When the optical density of the *A. tumefaciens* cells was 0.6 (OD_600nm_), the bacterial cells were pelleted and resuspended in infiltration solution (10 mM MgCl2, 10 mM MES, 20 µM 5-azacytidine, 0.5 mM ascorbic acid, 0.03% tween-20 and 150 mM acetosyringone, pH 5.6). The suspension was adjusted to an OD_600nm_ of 0.3. For the mixed infiltration, each suspension was adjusted to an OD_600nm_ of 0.6. The two suspensions were equally mixed before injection. The 6-week-old *N. benthamiana* plants to be used were infiltrated by using a needleless syringe. The infiltrated areas in the leaves were imaged at 3 d after infiltration with a confocal laser scanning microscope (Leica TCS SP8 MP) using a 40× water immersion lens.

## 5. Conclusions

Heterografts involving distantly related species provided us with a valuable offer to uncover macromolecule signaling in plants, particularly for those highly conserved RNAs. We used an *At/Nb* grafting system to identify six promising miRNAs that were moving from root to shoot, providing a foundation for understanding the shootward signaling mechanisms. Among them, *miR164* could work as a shootward signaling macromolecule to coordinate root and shoot development.

## Figures and Tables

**Table 1:** Deep sequencing of small RNA libraries from the *At* scion and mapping to the *At* and *Nb* genomes.

Samples	Species		Tissue Samples	Total Reads	Clean Reads (%)	Total sRNA Reads from *At*			
	Scion	Rootstock				sRNA Reads	Mapped sRNA Reads (%)	Unmapped sRNA Reads	Re-Mapping to *Nb* (%)
AGS	*A.thaliana*	*N.benthamiana*	cauline leaf, stem, flower	77,896,720	62,481,229 (80.21%)	41,991,400	25,206,466 (60.03%)	16,784,934	298,739 (0.71%)
ACS	*A.thaliana*	*A.thaliana*	cauline leaf, stem, flower	76,187,700	57,001,571 (74.82%)	34,161,421	20,766,148 (60.79%)	13,395,273	237,220 (0.69%)

**Table 2:** Deep sequencing of small RNA libraries from *Nb* rootstock and mapping to *At* and *Nb* genome.

Sample	Species		Tissue Samples	Total Reads	Clean Reads (%)	Total sRNA Reads from *Nb*			
	Scion	Rootstock				sRNA Reads	Mapped sRNA Reads (%)	Unmapped sRNA Reads	Re-Mapping to *At* (%)
NGR	*A.thaliana*	*N.benthamiana*	root	68,325,049	62,307,593 (91.19%)	52,616,678	36,897,829 (70.13%)	15,718,849	152,412 (0.29%)
NCR	*N.benthamiana*	*N.benthamiana*	root	256,050,792	223,052,992 (87.11%)	166,724,440	112,256,797 (67.33%)	54,467,643	89,082 (0.05%)

**Table 3:** Mobile *Nb* miRNAs identified from the *At* scion in an *At/Nb* heterograft.

Sequencing ID	miRNA Family	Mature miRNA Sequence	Length	AGS Read Counts			ACS Read Counts		
				AGS1	AGS2	AGS3	ACS1	ACS2	ACS3
conservative_Niben101Scf00647_2272 (*Nb-miR156 variant or Nb-miR156v*)	miR156	UGACAGAAGAGAGUGGGC	18	4	9	6	0	0	0
conservative_Niben101Scf00747_2488 (*Nb-miR164 variant or Nb-miR164v*)	miR164	UGGAGAAGCAGGGCACAUGC	20	1	1	1	0	0	0
conservative_Niben101Scf02279_7619 (*Nb-miR395-1*)	miR395	CUGAAGUGUUUGGGGGAACUCU	22	3	13	1	0	0	0
conservative_Niben101Scf02027_6631 (*Nb-miR1446 variant or Nb-miR1446v*)	miR1446	UUCUGAACUCUCUCCCUCAAU	21	0	3	0	0	0	0
conservative_Niben101Scf02778_9073 (*Nb-miR397 variant or Nb-miR397v*)	miR397	UCAUUGAGUGCAGCGUUGAUGA	22	1	3	5	0	0	0
conservative_Niben101Scf01112_4153 (*Nb-miR395-2*)	miR395	CUGAAGUGUUUGGGGGAACUCCG	23	25	47	45	0	0	0

## Data Availability

The small RNA deep sequencing data are available at the NCBI database under the accession ID PRJNA744705.

## Supplementary Materials

The following are available online at https://www.mdpi.com/article/10.3390/ijms222312821/s1.
