# Supplementary material for: Root-to-Shoot Long-Distance Mobile miRNAs Identified from Nicotiana Rootstocks
Source: Int J Mol Sci. 2021 Nov 26;22(23):12821. doi: 10.3390/ijms222312821 (PMC8657949; doi:10.3390/ijms222312821)
Supplement: Supplementary file 1 [file ijms-22-12821-s001.zip › ijms-1475480- supplementary/supplementary data for production/Figure S1.pdf]

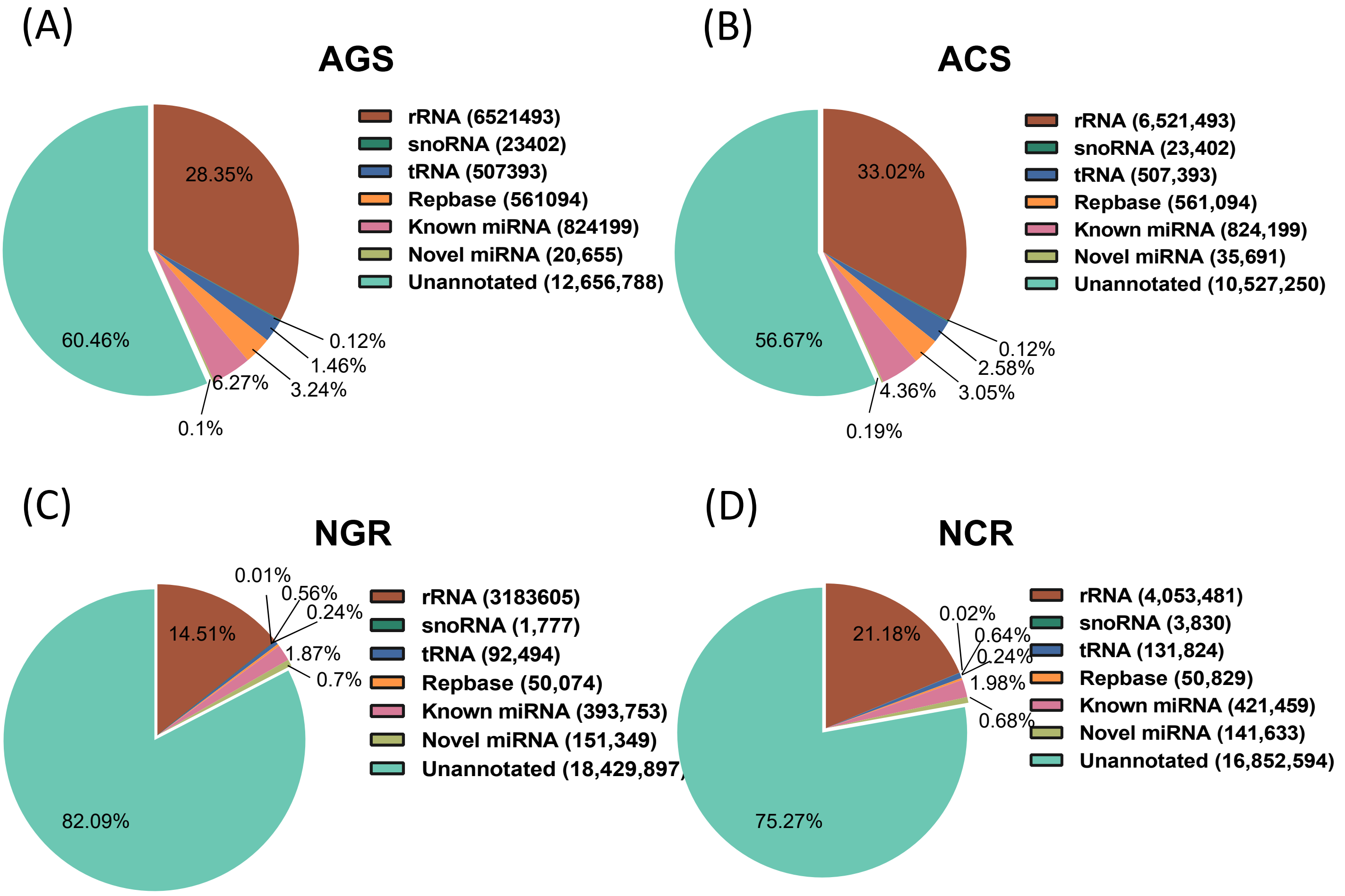

Figure S1. Small RNA classification. (A) Small RNA distribution in AGS library (*At*/*Nb* scion). (B) Small RNA distribution in ACS library (*At*/*At* scion). (C) Small RNA distribution in NGR library (*At*/*Nb* rootstock). (D) Small RNA distribution in NCR library (*Nb*/*Nb* rootstock).
