# Supplementary material for: Root-to-Shoot Long-Distance Mobile miRNAs Identified from Nicotiana Rootstocks
Source: Int J Mol Sci. 2021 Nov 26;22(23):12821. doi: 10.3390/ijms222312821 (PMC8657949; doi:10.3390/ijms222312821)
Supplement: Supplementary file 1 [file ijms-22-12821-s001.zip › ijms-1475480- supplementary/supplementary data for production/Figure S2-with caption.pdf]

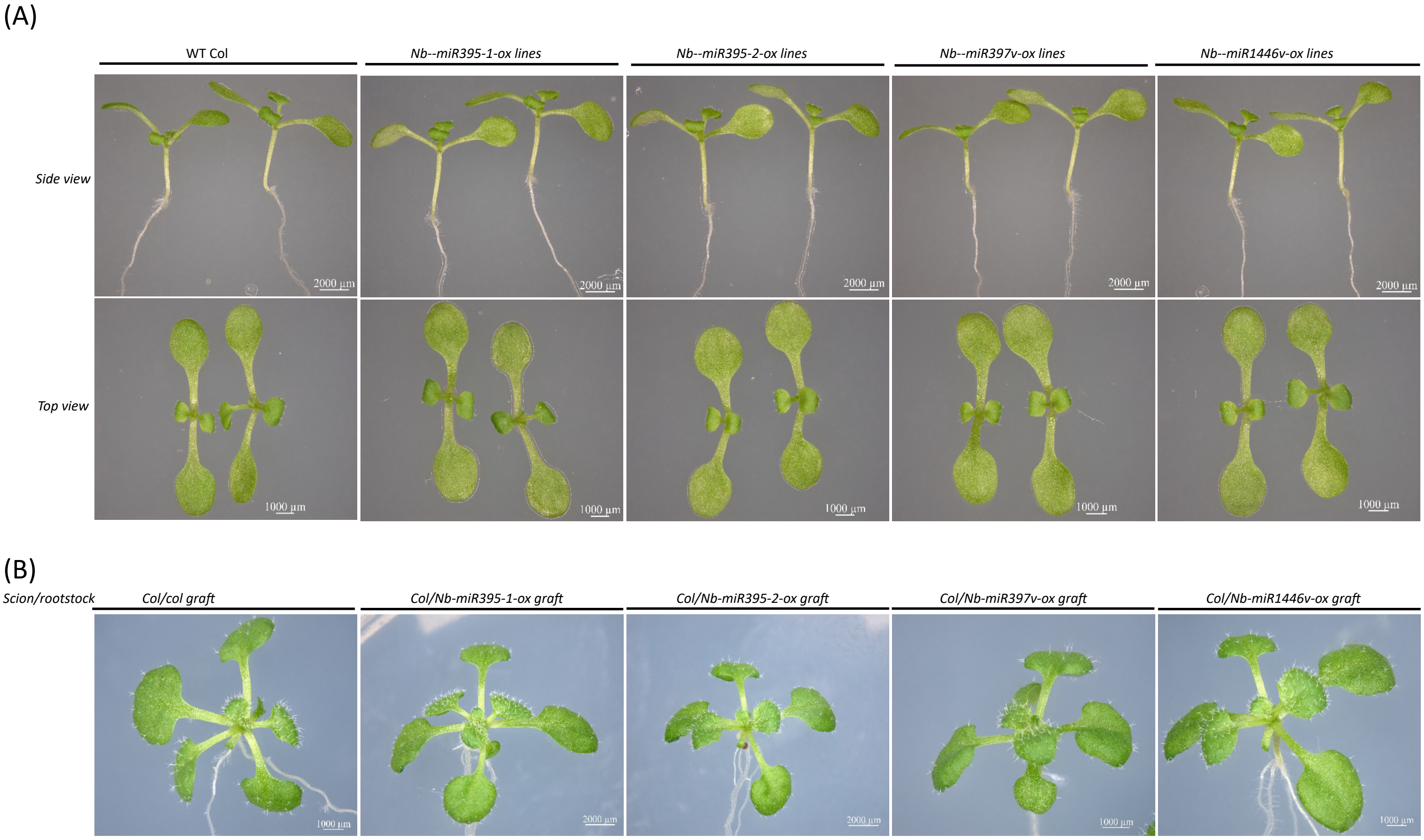

Figure S2. Growth phenotypes of *Arabidopsis* overexpressing lines of *Nb-miR395-1, Nb-miR395-2, Nb-miR397v* and *Nb-miR1446v* (A), and the grafting phenotye by using wild *Col-0* as scion and these overexpressing lines as rootstock respectively.
