# Supplementary material for: Root-to-Shoot Long-Distance Mobile miRNAs Identified from Nicotiana Rootstocks
Source: Int J Mol Sci. 2021 Nov 26;22(23):12821. doi: 10.3390/ijms222312821 (PMC8657949; doi:10.3390/ijms222312821)
Supplement: Supplementary file 1 [file ijms-22-12821-s001.zip › ijms-1475480- supplementary/supplementary data for production/Table S6.pdf]

Table S6. Primers used in this study.

Name	Sequence(5'-3')
**For Stem-loop**	
U6	GTGCAGGGTCCGAGGTTTTGGACCATTTCTCGAT
ath-miR158a-3p	GTCGTATCCAGTGCAGGGTCCGAGGTATTCGCACTGGATACGAC**TGCTTT**
ath-miR846-3p	GTCGTATCCAGTGCAGGGTCCGAGGTATTCGCACTGGATACGAC**AATTCA**
ath-miR398b-3p	GTCGTATCCAGTGCAGGGTCCGAGGTATTCGCACTGGATACGAC**CAGGGG**
ath-miR161.1	GTCGTATCCAGTGCAGGGTCCGAGGTATTCGCACTGGATACGAC**ACCCCG**
ath-miR156b-3p	GTCGTATCCAGTGCAGGGTCCGAGGTATTCGCACTGGATACGAC**ACTGAC**
ath-miR399a	GTCGTATCCAGTGCAGGGTCCGAGGTATTCGCACTGGATACGAC**CAGGGC**
ath-miR163	GTCGTATCCAGTGCAGGGTCCGAGGTATTCGCACTGGATACGA**CATCGAA**
Nb-miR395-1	GTCGTATCCAGTGCAGGGTCCGAGGTATTCGCACTGGATACGAC**AGAGTT**
Nb-miR395-2	GTCGTATCCAGTGCAGGGTCCGAGGTATTCGCACTGGATACGAC**CGGAGT**
Nb-miR156	GTCGTATCCAGTGCAGGGTCCGAGGTATTCGCACTGGATACGAC**GCCCAC**
Nb-miR397	GTCGTATCCAGTGCAGGGTCCGAGGTATTCGCACTGGATACGAC**TCATCA**
Nb-miR164	GTCGTATCCAGTGCAGGGTCCGAGGTATTCGCACTGGATACGAC**GCATGT**
Nb-miR1446	GTCGTATCCAGTGCAGGGTCCGAGGTATTCGCACTGGATACGAC**ATTGAG**
**For qRT-PCR**	
U6-F	GGAACGATACAGAGAAGATTAGCA
U6-R	GTGCAGGGTCCGAGGT
ath-miR158a-3p-F	GCGCGTCCCAAATGTAGAC
ath-miR158a-3p-R	AGTGCAGGGTCCGAGGTATT
ath-miR846-3p-F	GCGCGTTGAATTGAAGTGCT
ath-miR846-3p-R	AGTGCAGGGTCCGAGGTATT
ath-miR398b-3p-F	CGCGTGTGTTCTCAGGTCA
ath-miR398b-3p-R	AGTGCAGGGTCCGAGGTATT
ath-miR161.1-F	CGCGCGTGAAAGTGACTACAT
ath-miR161.1-R	AGTGCAGGGTCCGAGGTATT
ath-miR156b-3p-F	CGCGTGCTCACCTCTCTTTCT
ath-miR156b-3p-R	AGTGCAGGGTCCGAGGTATT
ath-miR399a-F	CGCGTGCCAAAGGAGATTT
ath-miR399a-R	AGTGCAGGGTCCGAGGTATT
ath-miR163-F	GCGTTGAAGAGGACTTGGAAC
ath-miR163-R	AGTGCAGGGTCCGAGGTATT
Nb-miR395-1-F	CGCTGAAGTGTTTGGGGG
Nb-miR395-1-R	AGTGCAGGGTCCGAGGTATT
Nb-miR395-2-F	CGCTGAAGTGTTTGGGGGA
Nb-miR395-2-R	AGTGCAGGGTCCGAGGTATT
Nb-miR156-F	CGCGCGTGACAGAAGAGA
Nb-miR156-R	AGTGCAGGGTCCGAGGTATT
Nb-miR397-F	GCGTCATTGAGTGCAGCGT
Nb-miR397-R	AGTGCAGGGTCCGAGGTATT
Nb-miR164-F	GCGTGGAGAAGCAGGGC

Nb-miR164-R	AGTGCAGGGTCCGAGGTATT
Nb-miR1446-F	GCGCGTTCTGAACTCTCTCC
Nb-miR1446-R	AGTGCAGGGTCCGAGGTATT

For overexpression

Nb-miR395-1-ox-F	CTGATTAACAGCTCGCAATTGCCAAGTTCAATCCGATCAAAATG
Nb-miR395-1-ox-R	CTTACTCAGTTAGGTCTACAGGAACCTGATCAATAGCATATACG
Nb-miR395-2-ox-F	CTGATTAACAGCTCGCAATTGGGGTAAGCTGTCTATATCACCCCT
Nb-miR395-2-ox-R	CTTACTCAGTTAGGTCACTAAGCTCCCTTCGAAATTCCA
Nb-miR397-ox-F	CTGATTAACAGCTCGCAATTGGGTGCACATTACAGGTTCAAATCT
Nb-miR397-ox-R	CTTACTCAGTTAGGTCAAAATGTACTACTTGAAAGCTCTAATGTACTAT
Nb-miR164-ox-F	CTGATTAACAGCTCGCAATTGATTTTACGCTAACCATCAACCTACTAA
Nb-miR164-ox-R	CTTACTCAGTTAGGTCAGTCATTCAAGATTGATTCTTCTAAATAGC
ath-miR163-ox-F	CTGATTAACAGCTCGCAATTGAGCATAGGTCTTGATTGGTGGAA
ath-miR163-ox-R	CTTACTCAGTTAGGTCACCTAGAAACCCATATTTTCAGGC

For RT-PCR

Nb-miR395-1-ox-F1	CTGATTAACAGCTCGCAATTGCCAAGTTCAATCCGATCAAAATG
Nb-miR395-1-ox-R1	CTTACTCAGTTAGGTCTACAGGAACCTGATCAATAGCATATACG
Nb-miR395-1-ox-F2	TTCCCCCTAGAGTTCTCCTGA
Nb-miR395-1-ox-R2	CAGAGTTCCCCCAAACACTTC
Nb-miR395-2-ox-F1	CTGATTAACAGCTCGCAATTGGGGTAAGCTGTCTATATCACCCCT
Nb-miR395-2-ox-R1	CTTACTCAGTTAGGTCACTAAGCTCCCTTCGAAATTCCA
Nb-miR395-2-ox-F2	GGTTTCGCCTAGAGTTCTCCT
Nb-miR395-2-ox-R2	CGGAGTTCCCCCAAACAC
Nb-miR164-ox-F1	CTGATTAACAGCTCGCAATTGATTTTACGCTAACCATCAACCTACTAA
Nb-miR164-ox-R1	CTTACTCAGTTAGGTCAGTCATTCAAGATTGATTCTTCTAAATAGC
Nb-miR164-ox-F2	TTTCGAAGCGCCTTAAACAG
Nb-miR164-ox-R2	TTAGCATGTGCCCTGCTTCT
ath-miR163-ox-F1	CTGATTAACAGCTCGCAATTGAGCATAGGTCTTGATTGGTGGAA
ath-miR163-ox-R1	CTTACTCAGTTAGGTCACCTAGAAACCCATATTTTCAGGC
ath-miR163-ox-F2	TGCGCAGTGCTTAAATCGTA
ath-miR163-ox-R2	CCATATTTTCAGGCACAACC

For confocal

Nb-miR395-1-SL24-F1	TAATTCTCGAGTGTGCCCAAGTTCAATCCGATCAAAATG
Nb-miR395-1-SL24-R1	CTTACTCAGTTAGGTCTACAGGAACCTGATCAATAGCATATACG
Nb-miR395-1-SL24-F2	TAATTCTCGAGTGTGCTTCCCCCTAGAGTTCTCCTGAAT
Nb-miR395-1-SL24-R2	CTTACTCAGTTAGGTCCAGAGTTCCCCCAAACACTTCA
Nb-miR397-SL24-F1	TAATTCTCGAGTGTGCGGTGCACATTACAGGTTCAAATCT
Nb-miR397-SL24-R1	CTTACTCAGTTAGGTCAAAATGTACTACTTGAAAGCTCTAATGTACTAT
Nb-miR397-SL24-F2	TAATTCTCGAGTGTGCTTTTCATCAACGCTGCACTCA
Nb-miR397-SL24-R2	CTTACTCAGTTAGGTCCGATGATTCGATTCCTATATTAACAAA
Nb-miR164-SL24-F1	TAATTCTCGAGTGTGCATTTTACGCTAACCATCAACCTACTAA

Nb-miR164-SL24-R1	CTTACTCAGTTAGGTCAGTCATTCAAGATTGATTCTTCTAAATAGC
Nb-miR164-SL24-F2	TAATTCTCGAGTGTGCTTTCGAAGCGCCTTAAACAGA
Nb-miR164-SL24-R2	CTTACTCAGTTAGGTCTTAGCATGTGCCCTGCTTCTC
GUS-SL24-F	TAATTCTCGAGTGTGCAATTGGTGTGAACAACGAACTGAACTGGC
GUS-SL24-R	CTTACTCAGTTAGGTCACTGCCACTGACCGGATGC
FT-SL24-SL24-F	AATTCTCGAGTGTGCACAAATTAAAGAAGCAGAAAC
FT-SL24-SL24-R	TTACTCAGTTAGGTCAATTGATAGGCATCATCACCGTTC
**For Golden Gate Cloning**	
Nb-miR164 P1	TAATTCTCGAGTGTGCGGTCTCTGGAGAAGCAGGGCACATGCGTGTGAACAACGAACTGAAC
Nb-miR164 P2	GGTCTCTCCGTTAAAGAAATCATGGAAGTA
Nb-miR164 P3	GGTCTCCGGATGGAGAAGCAGGGCACATGCCTATGCCGGAATCCATCGCA
Nb-miR164 P4	GGTCTCTCCGGTGGTTACAGTCTTGCGCGA
Nb-miR164 P5	GGTCTCCGGATGGAGAAGCAGGGCACATGCGCGTCTGTTGACTGGCAGGT
Nb-miR164 P6	GGTCTCTCCGTCCCGCTAGTGCCTTGTCCA
Nb-miR164 P7	GGTCTCCGGATGGAGAAGCAGGGCACATGCCTTTGCAAGTGGTGAATCCG
Nb-miR164 P8	CTTACTCAGTTAGGTCGGTCTCGGGTAGATATCACACTCTGT
